# Socio-Motor Improvisation in Schizophrenia: A Case-Control Study in a Sample of Stable Patients

**DOI:** 10.3389/fnhum.2021.676242

**Published:** 2021-10-21

**Authors:** Robin N. Salesse, Jean-François Casties, Delphine Capdevielle, Stéphane Raffard

**Affiliations:** ^1^University Department of Adult Psychiatry, Montpellier University Hospital, Montpellier, France; ^2^CTIsuccess by Mooven, Contract Research Organisation, Montpellier, France; ^3^INSERM U1061, Neuropsychiatrie Recherche Épidémiologique et Clinique, Université de Montpellier, Montpellier, France; ^4^Epsylon Laboratory EA 4556, University Paul Valéry Montpellier 3, Montpellier, France

**Keywords:** psychiatry, embodiment, coordination dynamics, human bonding, mirror game

## Abstract

Improvising is essential for human development and is one of the most important characteristics of being human. However, how mental illness affects improvisation remains largely unknown. In this study we focused on socio-motor improvisation in individuals with schizophrenia, one of the more debilitating mental disorder. This represents the ability to improvise gestures during an interaction to promote sustained communication and shared attention. Using a novel paradigm called the mirror game and recently introduced to study joint improvisation, we recorded hand motions of two people mirroring each other. Comparing Schizophrenia patients and healthy controls skills during the game, we found that improvisation was impaired in schizophrenia patients. Patients also exhibited significantly higher difficulties to being synchronized with someone they follow but not when they were leaders of the joint improvisation game. Considering the correlation between socio-motor synchronization and socio-motor improvisation, these results suggest that synchronization does not only promote affiliation but also improvisation, being therefore an interesting key factor to enhance social skills in a clinical context. Moreover, socio-motor improvisation abnormalities were not associated with executive functioning, one traditional underpinning of improvisation. Altogether, our results suggest that even if both mental illness and improvisation differ from normal thinking and behavior, they are not two sides of the same coin, providing a direct evidence that being able to improvise in individual situations is fundamentally different than being able to improvise in a social context.

## Introduction

Improvisation is commonly considered to be akin to insanity (Barrantes-Vidal, 2004). Famous so-called *mad genius* Syd Barrett, early lead singer, guitarist and principal songwriter behind the rock band *Pink Floyd*, or John Nash, the father of *game theory* and Nobel Prize in economics for “The pioneering analysis of equilibria in the theory of non-competitive games,” are exceptional cases of improvisers diagnosed with schizophrenia. However, only a small group of people with schizophrenia are able to walk their impairment and benefit from it, the majority being left with misunderstanding and stigmatization (Thornicroft et al., 2009), Social impairment is a major symptom shared by mental illnesses (Giacco et al., 2012; Kidd, 2013), affecting patients functioning, outcomes and quality of life. Abnormal, “bizarre” motor behaviors (“weird” posturing or aimless movements), and deficits in socio-motor synchronization (“the natural tendency to synchronize gestures during a social interaction”), were considered as characteristic of some individuals with schizophrenia, leading to a lack of rapport and feelings of connectedness with these patients (Bleuler, 1950; Varlet et al., 2012). Whereas socio-motor synchronization capture to what extend people act within the same timing, we hypothesize that socio-motor improvisation, the ability to improvise timely and new gestures during a social interaction, is a necessary property for promoting sustained communication and shared attention. We propose here to study this timely improvisation in individuals with schizophrenia through the scope of socio-motor improvisation and socio-motor synchronization.

Dance improvisation between multiple dancers is an excellent example of collaborative motor improvisation. New ideas do not appear simply from the internal processes of a single person but from the interaction within a group at the level of the minds, bodies and environment (Łucznik, 2015). Much human improvisation arises from activities that take place during social interactions. Encompassing behavioral outcomes in a social context, socio-motor improvisation refers to creative actions performed by two or more people during a social interaction (Noy et al., 2011; Gueugnon et al., 2016). It is fundamental here for a sake of understanding to remark that a successful improvisation in a collaborative context needs individuals to be connected, synchronized, at some point (Bernieri and Rosenthal, 1991; Noy et al., 2011). Socio- motor behavior constitutes a strong and necessary conveyor of social skills, synchrony playing a fundamental role during communicative non-verbal behaviors (Hove and Risen, 2009; Schmidt et al., 2012). Being synchronized is therefore also necessary to characterize this ability to create novelty together. Socio-motor improvisation and synchronization are the two sides of a same coin. Defined by Bernieri and Rosenthal (1991) as the smooth meshing in time of the simultaneous rhythmic activity of two people, synchrony predicts subsequent affiliation ratings (Hove and Risen, 2009), increases rapport (Bernieri, 1988) and cooperation (Wiltermuth and Heath, 2009) between individuals. Thus, socio-motor synchronization plays a fundamental role in enhancing connectedness, social rapport or cohesion in human interactions (Marsh et al., 2009; Chartrand and Lakin, 2013). Following Orth et al. (2017), rather than referring to behaviors that are uniquely generated by a cognitive system, socio-motor improvisation describes unfolding actions that are original because socio-motor improvisation relative to the individual or group, and functional because they support task success in terms of socio-motor synchronization (Hristovski et al., 2011).

Reducing the definition of improvisation to its *cognitive* dimension also affects schizophrenia studies and the understanding of this disorder, focusing on prototypical psychotic symptoms such as incoherent thinking, loss of contact with reality, hallucinations and cognitive deficits (Mier and Kirsch, 2015). Schizophrenia is a common psychotic illness and is considered one of the leading causes of disability in rich countries. Schizophrenia is often characterized by its psychotic symptoms and cognitive deficits, but social interaction deficits remain one of its most significant, defining, debilitating and tremendous features (Giacco et al., 2012; Kidd, 2013). Social interaction deficits affect patients’ long-term functioning, outcomes and quality of life but are partially explained by clinical symptom severity (Schmidt et al., 2011). Recent interdisciplinary research highlighted the huge impact of implicit non-verbal motor behaviors on social exchanges, opening interesting ways to study and understand mental illnesses in their social context and complexity (Del-Monte et al., 2013; Lavelle et al., 2013, 2014). Many ecologically valid paradigms of gesture use demonstrates that patients with schizophrenia are truly impaired when imitating hand gestures, which is actually linked to social functioning and motor abnormalities (e.g., Matthews et al., 2013; Walther et al., 2015, 2016). Varlet et al. (2012) assessed non-verbal motor interaction comparing healthy and schizophrenia patients when performing socio-motor synchronization tasks. They showed that unintended synchronization was preserved while voluntary synchronization was impaired, interpreted as a potential effect of executive dysfunctioning. Confirming results were obtained using priming procedures (Raffard et al., 2015) and virtual social environments (Raffard et al., 2018) that positively affected unintended socio-motor synchronization in schizophrenia patients. Previous research on healthy subjects has shown that the emergence and stability of such unintended or intended synchronization is closely associated with affiliation, and social cohesion (Bernieri, 1988; Hove and Risen, 2009; Schmidt et al., 2011).

Studies regarding improvisation in mental illness have mainly focused their interest on cognition, representing to what extend executive abilities such as updating, shifting and inhibition (Benedek et al., 2014) are preserved in mental illnesses. However, one may consider improvisation in its social context, that is during joint interaction with others. Therefore, improvisation refers to an individual’s ability to respond in a divergent, original or flexible manner to motor challenges induced by social interaction. Noy et al. (2011) introduced the mirror game as a paradigm for studying the dynamics of two people improvising motion together. Interestingly, the mirror game is based on an ambiguity between two contradictory instructions: socio-motor improvisation and socio-motor synchronization. Indeed, improvising implies the production of “various, complex and interesting” movements but risking being poorly synchronized due to the unpredictability of the motion performed. At the contrary but in a same time, being synchronized implies the production of highly predictable simultaneous and coordinated motion but obviously risking decreasing the quality and the level of the improvisation. Solving this trade-off between improvisation and synchronization in the mirror game reflects the core of our experimental protocol. In the present study, we combined, on the one hand, human movement paradigms specifically dedicated to study non-verbal bodily social interactions (Noy et al., 2011) and on the other hand, well-known psychological measures of cognitive functioning associated with cognitive improvisation (Barrantes-Vidal, 2004). Hence, we investigated whether schizophrenia influences socio-motor improvisation. Due to impairments in voluntary but not unintended socio-motor synchronization tasks (Varlet et al., 2012), we expect that individuals with schizophrenia would perform poorly both in terms of socio-motor improvisation and socio-motor synchronization than healthy participants. Additionally, social science studies demonstrated that the motor system influences cognition, and that cognition also affects bodily actions, we therefore expected positive correlation between *cognitive* improvisation and socio-motor improvisation.

## Materials and Methods

### Participants

We included 60 participants: 30 schizophrenia stable outpatients and 30 age-matched healthy participants (Table 1). Patients were recruited from the University Department of Adult Psychiatry and fulfilled the Diagnostic and Statistical Manual of Mental Disorders (American Psychiatric Association, 2000) criteria for schizophrenia. Diagnoses were established using the Structured Clinical Interview (SCID) for DSM–IV-TR. All patients received anti-psychotic medication. Exclusion criteria for both the clinical and non-clinical groups were: (a) known neurological disease, (b) Axe II diagnosis of developmental disorders or (c) substance abuse in the past month.

**TABLE 1:** Socio-demographic description and mean, standard deviation, median and range of values are presented for the age, level of education, premorbid IQ (fNART), neurological soft signs (NSS), positive and negative symptom scale (PANSS), medication (chlorpromazine equivalent dose CPZE in mg/day), gender, trail making test (TMT A, TMT B, TMT B-A), global functioning (EGF), liebowitz social anxiety scale (LSAS anxiety, LSAS avoidance), social cognition and theory of mind (ToM15 false belief, ToM15 understanding).

	**Schizophrenia patients (*N* = 30)**				**Healthy controls (*N* = 28)**				**Statistics**			
	**M**	**SD**	**Med**	**Rg**	**M**	**SD**	**Med**	**Rg**				
Age	33.8	10.4	32.5	[18–58]	31.6	5.7	30.5	[24–49]	*U* = 391.5		*z* = −0.44	*p* = 0.66
Level of education	4.4	1.1	4	[3–6]	4.4	0.7	4	[3–6]	*U* = 401.5		*z* = −0.31	*p* = 0.76
fNart	22	7	22.0	[7–33]	23.3	4.3	23.5	[15–30]	*U* = 369.5		*z* = −0.79	*p* = 0.43
NSS	16.6	7	16.1	[6.9–34.5]	8.2	2.2	8	[4–12.7]	*U* = 90.5		*z* = −5.02	***p* = 0.00**
**PANSS**												
Positive	9.4	2.4	9	[7–15]								
Negative	15.1	7.1	13.0	[7–33]								
General psychopathology	27.2	5.6	27.5	[19–38]								
Total score	51.8	11	50.0	[35–75]								
PANSS-Q10	1.9	1.3	1	[1–5]								
CPZE	533	406	400	[150–2000]								
	N	%			N	%						
Gender/*Man*	28	93			25	89			χ^2^(1, N) = 0.30			*p* = 0.58
TMT A	35.5	11.7	34.0	[20–68]	27.8	7.6	26.5	[12–45]	*U* = 252.0	*z* = −2.45		***p* = 0.01**
TMT B	102	59.6	91.0	[34–300]	67.9	32.0	58	[34–195]	*U* = 189.5	*z* = −3.45		***p* = 0.00**
TMT B-A	66.5	55.7	52.0	[14–259]	40.1	30.5	32.5	[11–169]	*U* = 224.5	*z* = −2.89		***p* = 0.00**
EGF	43.8	8.1	42	[25–61]	79.3	8.7	80.5	[61–95]	*U* = 0.5	*z* = 6.52		***p* = 0.00**
LSAS anxiety	21.6	14.3	20	[0–58]	13.3	8	12.5	[1–36]	*U* = 269.5	*z* = −2.33		***p* = 0.00**
LSAS avoidance	17.5	11.8	20	[0–39]	9.3	7.3	7.5	[1–25]	*U* = 238.5	*z* = −2.82		***p* = 0.02**
Social cognition	21	5.1	22.0	[10–30]	16.5	4	17	[4–27]	*U* = 210.5	*z* = −3.26		***p* = 0.00**
ToM15 false belief	10.5	2.6	11	[4–14]	12.6	2	13	[6–15]	*U* = 192.5	*z* = 3.53		***p* = 0.00**
ToM understanding	13.7	1.7	14.0	[9–15]	14.4	0.9	15	[11–15]	*U* = 340.5	*z* = 1.23		*p* = 0.22

*Group statistics are presented using appropriates non-parametric tests. Significant *p* values ¡ 0.05 are represented in bold.*

All participants were native French speakers with a minimal reading level validated using the National Adult Reading Test (f-NART, Mackinnon and Mulligan, 2005) and were able to perform the interaction task described below. All of the participants were adults with normal or corrected-to-normal vision. Age-matched healthy participants were recruited from a call for participation in the hospital’s website and the community. They had no lifetime history of any psychosis diagnoses according to the SCID. Two healthy participants were discarded from the study because they dropped out before the end of the experiment for personal reasons. All participants provided written informed consent, prior to the experiment approved by the National Ethics Committee (CPP Sud-Méditerranée-III, Nîmes, France, #2013.04.05 and DA/2013-136) and conforming to the Declaration of Helsinki. Accordingly with identifying information policies, written informed consent for publication of identifying information/images was obtained. The thirty schizophrenia patients composed the Schizophrenia group, and the twenty-eight matched control participants composed the Control group.

### Setup

**FIGURE 1:**
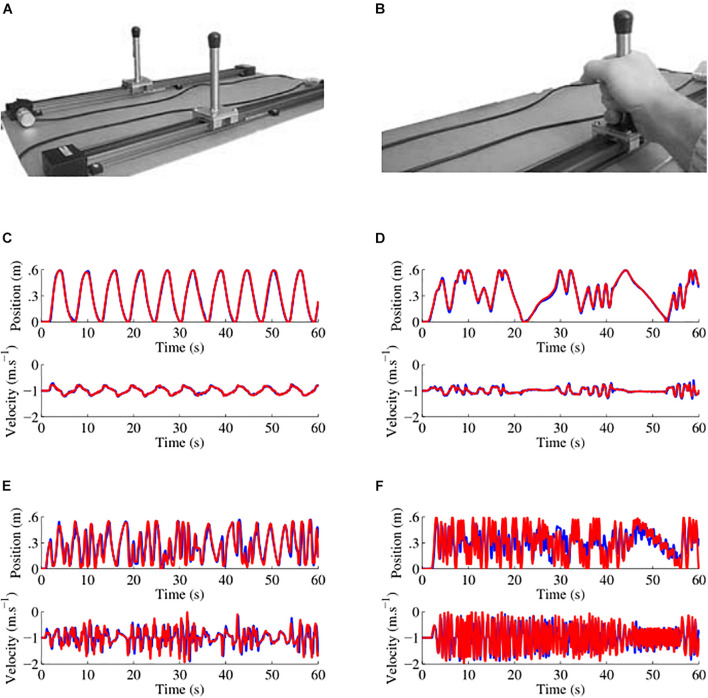
Custom hardware apparatus and examples of data collected. **(A)** Picture of the apparatus used to play the mirror game, the motion of two players moving handles along 0.6 m tracks was sampled at 100 Hz with a spatial resolution of 0.054 mm. **(B)** Handles were held with one or two hands depending on participant preference. **(C–F)** Examples of position and velocity traces for four different players in the first round where the participant led the game. Here the leader is the red player. In **(C)** the traces are those of a schizophrenia patient with a socio-motor improvisation of 0.037 and a socio-motor synchronization of 0.998. In **(D)** the traces are those of another schizophrenia patient with a socio-motor improvisation of 0.040 and a socio-motor synchronization of 0.984. In **(E)** the traces are those of a healthy control with a socio-motor improvisation of 0.092 and a socio-motor synchronization of 0.867. In **(F)** the traces are those of another schizophrenia patient with a socio-motor improvisation of 0.108 and a socio-motor synchronization of 0.716.

We designed and built a custom hardware similar to the one designed by Noy et al. (2011) for recording dyadic motions in the mirror game (Figure 1). Players moved handles along parallel tracks 0.6 m long. Handle positions were measured by an optical encoder with a spatial resolution of 0.054 mm and were sampled at 100 Hz. Data were recorded on a computer using National Instruments LABVIEW 13.0 software.

### Procedure

The experiment was completed in two visits. Visit 1 involved completing informed consent, clinical interviews and questionnaires. Visit 2 occurred approximately 1 or 2 days later. It consisted in a mirror game where participants and a confederate imitate each other in turns.

During visit 1, participants were rated with socio-demographic, psychological and psychopathology scales. Following recent recommendations from the social psychology literature (Doyen et al., 2012; Shanks et al., 2013), our participants were fully naive about the real goal of the experiment. To prevent any “un-blind experimenter effect,” both the experimenter and the confederate were fully naive about the diagnosis of participants and were not informed about the group participants belonged to (schizophrenia or healthy controls). All participants were asked to inform their age, gender, Level of Education. All participants were right-handed as assessed by informal verbal inquiry. They were also rated with the Neurological Soft Signs Scale (Krebs et al., 2000) to assess subtle abnormalities in sensory-perceptual, motor functions directly associated with schizophrenia pathology (Gupta, 1995; Walther and Strik, 2012) or induced by neuroleptic medications (D’Agati et al., 2012). Social cognition was measured using the Autism spectrum quotient questionnaire (Baron-Cohen et al., 2001) and a social false-belief test (Desgranges et al., 2012) to evaluate theory of mind. It refers to the ability to infer mental states (e.g., beliefs, desires, intentions, imagination, emotions) that cause behavior within a social environment. Finally, participants were rated with the trail making test (Delis et al., 2001). TMT is a validated tool to assess cognitive flexibility and has been widely used to assess cognitive improvisation (Moritz et al., 2002; Laere et al., 2018). In the schizophrenia group, we administered the Positive and Negative Syndrome Scale (Kay et al., 1987) using the Structured Clinical Interview for the PANSS (SCI-PANSS). The degree of interactional deficits was assessed through the PANSS “poor rapport” item Q10 (Riehle et al., 2015). During visit 2, participants were asked to play a mirror game with a confederate. The confederate was an expert in playing the mirror game in order to prevent any edge effect due to insufficient socio-motor skills. Players sat facing each other on either side of the experimental apparatus (Figures 1A,B). They hold the handles comfortably with one or both hands depending on their preference. First, the game and the procedure were explained to the players). Participant were given the instruction to “imitate each other, create synchronized and interesting motions, and enjoy playing together” (detailed description is given in Noy et al., 2011), and were reminded that the purpose was a cooperation to enjoy creating motions together and not a competition. The experimenter was present in the room during the game to give the instructions but did not intervene during the game. Second, participants practiced three warm-up 15 s rounds (Participant leader, Confederate leader, and Joint improvisation) to get acquainted with the procedure. In participant leader and confederate leader conditions, one player was leading the motion and the other was following. In joint-improvisation rounds, players moved together without a designated leader. Finally, they were asked to perform nine rounds of 60 s each. Rounds were separated with a 10 to 30 s pause to relax. According to the procedure proposed initially by Noy et al. (2011) rounds were counterbalanced in the following order: Participant leader, Confederate leader, Joint improvisation, Confederate leader, Joint improvisation, Participant leader, Joint improvisation, Participant leader, and Confederate leader.

### Data Reduction

Two socio-motor variables were extracted from the mirror-game: socio-motor improvisation and socio-motor synchronization. Position signals were pre-processed with MathWorks MATLAB Software. Position signals were interpolated with a shape-preserving Piecewise Cubic Hermite Interpolating Polynomial (PCHIP) to correct small variations in the sampling rate (Kahaner et al., 1989) and filtered with a zero-phase forward and reverse digital second-order low-pass (10 Hz cut-off) Butterworth filter. Position time-series were then used to numerically estimate their corresponding velocity time-series using a fourth-order finite difference scheme (Słowiński et al., 2016). A detailed sample of time series is displayed in Appendix Figure 1. Time-series performed by participants were mostly non-stationary, multi-modal and multi-scale due to the nature of the task. Capturing socio-motor improvisation therefore implies a measure of the complexity of the signal. Whereas usual measures of complexity signals refer to the entropy such as the Shannon entropy, the non-stationarity of the time-series collected needed the use of multiscale entropy measures. According to Noy et al. (2011), socio-motor improvisation was computed with a wavelet-based complexity measure. This measure is based on a wavelet decomposition, a numerical approach decomposing signals in building blocks localized in space and frequency. Therefore, the wavelet complexity of a signal was defined as the inverse of the number of wavelets needed to describe 95% of the signal. Following Noy et al. (2011), the socio-motor improvisation measure was the compression ratio: the number of coefficients used to achieve the reconstruction normalized by the number of samples in the round. The higher this ratio, the more complex the signal, indicating a higher improvisation. Socio-motor synchronization was calculated with the index of synchronization provided by Rosenblum et al. (1996). This index takes into account fluctuations in coordination over short time scales, for example within a movement cycle. This index is based on the circular variance of the relative phase estimated with the Hilbert transform method. It is generally defined as 1 minus the circular variance. The variance is a measure of dispersion, of stability of a variable. A variance close to 0 indicates that all values are identical and a variance of 1 indicates a strong dispersion between values. The synchronization index thus provides a value between 0 (no synchronization) and 1 (perfect synchronization).

### Statistical Analyses

Prior conducting statistical analyses, the three rounds in each condition were averaged according to the initial procedure proposed by Noy et al. (2011). Socio-motor improvisation and socio-motor synchronization were analyzed using two-way ANOVAs with Group (Schizophrenia vs. Healthy controls) as Group factor, with repeated measures on Conditions (Participant leader, Joint improvisation, Confederate leader). For all ANOVAs, Newman–Keuls *post hoc* tests were computed when the nature of the effects had to be specified. Size effects are reported using the partial eta squared *η_p_^2^* (Bakeman, 2005) and interpreted according to Cohen’s D (Cohen, 1988), where 0.02 corresponds to a small effect, 0.13 to a medium effect and 0.26 to a large effect. When necessary, alpha value of significance was corrected using the Bonferroni procedure. Questionnaires were separately compared for the schizophrenia and the control groups with a non-parametric U-Mann-Whitney test or a χ2 test for binary variables (e.g., gender). Pairwise comparisons between groups were performed using a *t*-test when necessary. The level of significance was set to *p* < 0.05 and was corrected using the Bonferroni procedure when necessary. Finally, we performed correlations to demonstrate the trade-off between socio-motor improvisation and socio-motor synchronization, and the potential links between *cognitive* improvisation and socio-motor improvisation. The level of significance was lowered for multiple comparisons using the Bonferroni procedure, corresponding alpha values are reported in the captions.

## Results

### Socio-Motor Performance

The mirror game allows capturing both socio-motor improvisation and socio-motor synchronization. Improvising implies the production of “various, complex and interesting” movements but risking being poorly synchronized. At the same time, being synchronous implies the production of simultaneous motion but risking decreasing the improvisation. Solving this trade-off between improvisation and synchronization in the mirror game reflects the core of our experimental protocol. Detailed kinematic characteristics are compared in Appendix Figures 2, 3.

**FIGURE 2:**
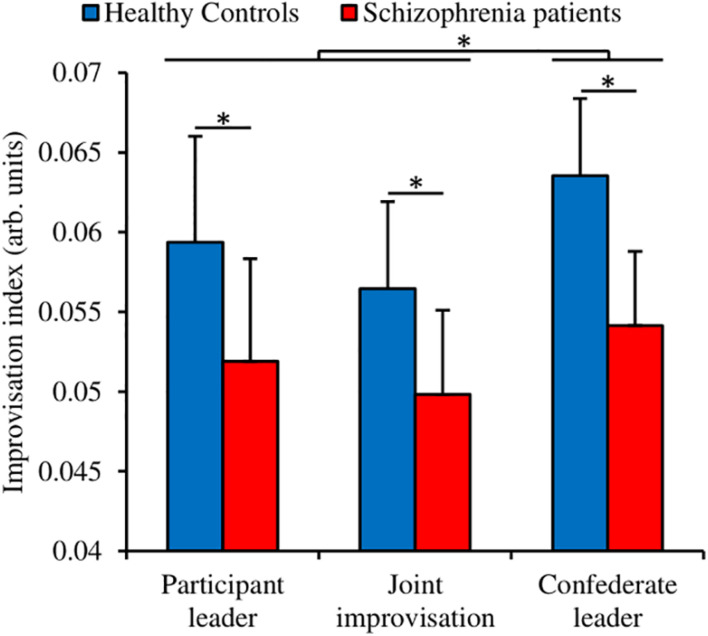
Socio-motor improvisation extracted from the participants motion. Error bars correspond to the standard deviation around the mean. Star indicates significant *post hoc* differences.

**FIGURE 3:**
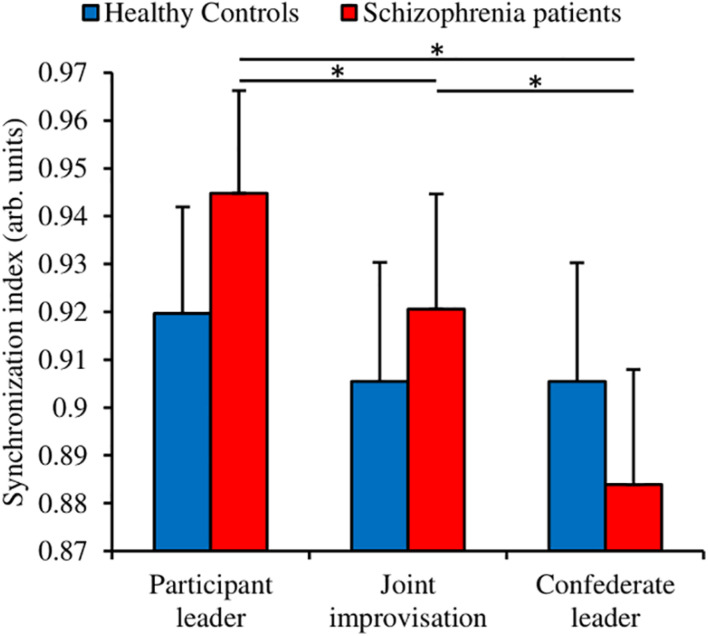
Socio-motor synchronization index extracted from the participants motion. Error bars correspond to the standard deviation around the mean. Star indicates significant *post hoc* differences.

#### Improvisation Schizophrenia Patients Improvise Less Than Healthy Controls

We measured the socio-motor improvisation with a wavelet transform decomposition (Noy et al., 2011). We performed a Group (Schizophrenia patients; Healthy controls) × Condition (Participant leader; Joint improvisation; Confederate leader) ANOVA with repeated measures on the Condition factor (Figure 2). The analysis revealed a significant small to medium Group effect [*F*(1,56) = 4.96, *p* = 0.030, *η_p_^2^* = 0.081] indicating that patients improvise less than healthy controls (respectively 0.052 vs. 0.060 in arbitrary units). The analysis also showed a significant small to medium Condition effect [*F*(2,112) = 6.65, *p* = 0.002, *η_p_^2^* = 0.106]. The Newman-Keuls *post hoc* decomposition of this effect revealed that the Confederate leader condition exhibited a higher degree of improvisation than the two others conditions (Participant leader = 0.056; Joint improvisation = 0.053; Confederate leader = 0.059). The analysis failed to reveal a Group × Condition interaction [*F*(2,112) = 0.44, *p* = 0.64, *η_p_^2^* = 0.008]. Altogether, these results suggest that healthy controls are more able to improvise than schizophrenia patients during socio-motor improvisation.

#### Synchronization Schizophrenia Patients Synchronize Better When Leading Than Following

We measured the socio-motor synchronization with the index of synchronization (Rosenblum et al., 1996). We performed a Group (Schizophrenia patients; Healthy controls) × Condition (Participant leader; Joint improvisation; Confederate leader) ANOVA with repeated measures on the Condition factor (Figure 3). The analysis failed to show a significant Group effect [*F*(1,56) = 0.20, *p* = 0.656, *η_p_^2^* = 0.004]. However, the analysis revealed a significant medium to large Condition effect [*F*(2,112) = 11.37, *p* = 0.0000, *η_p_^2^* = 0.169], and a significant small to medium Group × Condition interaction [*F*(2,112) = 4.86, *p* = 0.009, *η_p_^2^* = 0.080]. The Newman-Keuls *post hoc* decomposition of this interaction revealed that the schizophrenia group performed better when leading the game rather than improvising or following. Nevertheless, healthy controls were not affected by the conditions, performing equally in the three situations.

#### Socio-Motor Improvisation Versus Socio-Motor Synchronization

**FIGURE 4:**
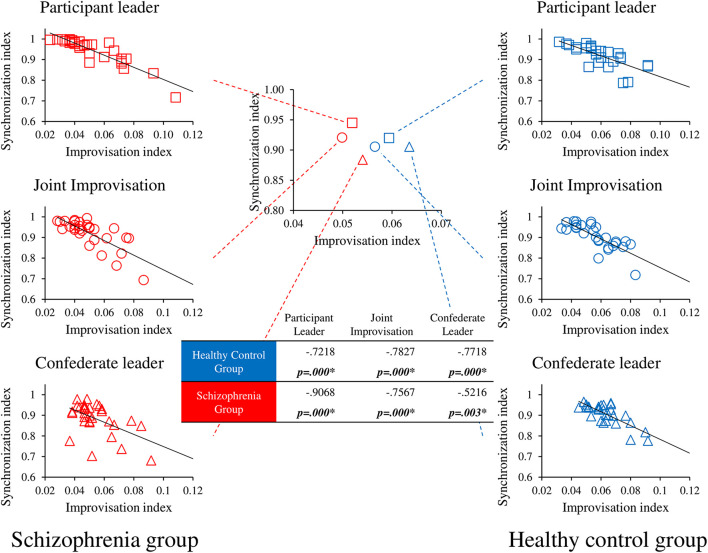
Correlational analyses between socio-motor improvisation and socio-motor synchronization. *r* and *p* values are presented in the table for the correlation between Socio-motor improvisation and Socio-motor synchronization for each group in the three conditions. *p* values < 0.1 are represented in bold and * represents significant correlations <0.05. Central figure represents the improvisation-synchronization trade-off for each group in each condition whereas peripheral figures details correlations for each condition in the schizophrenia group on the left side and the healthy control group on the right side.

We performed correlational analyses between socio-motor improvisation and the socio-motor for both groups (Schizophrenia patients, Healthy controls) in each of the three experimental conditions (Participant leader, Joint improvisation, Confederate leader). Results are represented in Figure 4. Socio-motor improvisation negatively correlates with socio-motor synchronization for both groups in all conditions, demonstrating the contradiction between improvising and being synchronized.

### Correlational Analyses

#### Symptomatology and Socio-Motor Performance

To better understand the above-mentioned results, we conducted a correlational analysis between the PANSS and socio-motor variables (Table 2). The analyses failed to reveal any significant correlations.

**TABLE 2:** Correlation between symptomatology and socio-motor variables for the schizophrenia group.

	**Socio-motor creativity**			**Socio-motor synchronization**		
	**Participant**	**Joint**	**Confederate**	**Participant**	**Joint**	**Confederate**
	**Leader**	**Improvisation**	**Leader**	**Leader**	**Improvisation**	**Leader**
**PANSS**						
Positive	0.0992	0.1403	0.0791	−0.1352	−0.2614	−0.3526
	*p* = 0.602	*p* = 0.460	*p* = 0.678	*p* = 0.476	*p* = 0.163	*p* = 0.056
Negative	0.0365	−0.0389	−0.1277	0.0148	−0.1745	−0.3348
	*p* = 0.848	*p* = 0.838	*p* = 0.501	*p* = 0.938	*p* = 0.357	*p* = 0.071
General psychopathology	−0.2748	−0.2373	−0.2481	0.24	−0.1017	−0.1229
	*p* = 0.142	*p* = 0.207	*p* = 0.186	*p* = 0.201	*p* = 0.593	*p* = 0.518
Total score	−0.095	−0.1151	−0.1911	0.1021	−0.2215	−0.3552
	*p* = 0.618	*p* = 0.545	*p* = 0.312	*p* = 0.591	*p* = 0.240	*p* = 0.054
PANSS Q-10	0.1509	0.0728	−0.092	−0.1338	−0.2801	−0.3087
	*p* = 0.426	*p* = 0.702	*p* = 0.629	*p* = 0.481	*p* = 0.134	*p* = 0.097
NSS	0.3258	0.203	0.1764	−0.2478	0.0005	−0.2543
	*p* = 0.079	*p* = 0.282	*p* = 0.351	*p* = 0.187	*p* = 0.998	*p* = 0.175
CPZE	0.1195	0.1474	0.0057	−0.0733	−0.3582	−0.0688
	*p* = 0.529	*p* = 0.437	*p* = 0.976	*p* = 0.700	*p* = 0.052	*p* = 0.718

**r* and *p* values are presented for the correlation between PANSS total score and its sub-dimensions, neurological soft signs NSS, medication (Chlorpromazine equivalent dose CPZE) and socio-motor improvisation and synchronization for each condition. Bonferroni’s adjustment for 6 comparisons lowers the alpha value from 0.05 to 0.0083.*

#### Cognitive Creativity and Socio-Motor Variables

Finally, we explored whether the exhibited deficit on socio-motor improvisation might be explained by a general deficit in executive functions and cognitive creativity. Result showed that cognitive creativity (TMT), AQ, TOM15 (false-belief) and socio-motor improvisation are not correlated (Appendix Table 1).

## Discussion

Improvising is often considered a trait that everyone possesses to come up with new and useful ideas. Whereas psychology and neuroscience researchers are identifying thinking processes and brain regions involved in *cognitive* improvisation, little is known about motor improvisation during social interactions such as dance or sport (Memmert and Roth, 2007; Memmert et al., 2010). Indeed, leisure activities such as team sports, dance, music or theatre are often described as highly improvising demanding, particularly in terms of group improvisation (Hart et al., 2014). They imply processes such as improvisation but also synchronization and togetherness in order to reach a common collective goal. The analogy with daily lives is outstanding since not only ideas might need to be improvised, but behaviors in a social context also. Because mental illnesses involves both cognitive and socio-behavioral impairments, the aim of this study was to focus on the ability to generate gestures during a social interaction, comparing healthy people with schizophrenia patients. Even if improvisation in motor actions does not pop-up in mind at first glance, it has been studied for decades in experimental psychology, human movement science and neuroscience under the capability of individuals to show original and functional motor actions as a fundamental aspect of skill, adaptability and flexibility (Seifert et al., 2016). From this perspective, socio-motor improvisation is obviously a cornerstone of social functioning. This originality in performing motor actions is observed at different levels: within individual, inter-individual and social levels. We assume that these levels are inter-dependent, social improvisation emerging from interactions observed at the individual level and between individuals. Therefore, our study proposes from a clinical perspective an understanding of how improvisation shapes social interactions and their deficits. We asked healthy controls and schizophrenia patients to play a mirror game with an expert player but naive about participants’ diagnosis. The goal was to “imitate each other, create synchronized and interesting motions, and enjoy playing together.” Participants role during the game was either leader, follower or uninstructed (Noy et al., 2011). We measured socio-motor improvisation, socio-motor synchronization and clinical variables.

Schizophrenia patients displayed impaired socio-motor improvisation compared to the healthy controls group in all conditions. Both groups exhibited, however, the same pattern of results, exhibiting a higher socio-motor improvisation when following an expert rather than leading or improvising. This result is perfectly in line with a recent research showing similar effects with sensibly lower values, presumably due to small differences in the apparatus used to capture the motion of the players (Brezis et al., 2017). Socio-motor improvisation being measured at the level of the dyad during a social game, patients were able to take advantage of the expert improvisational skill but to a lesser degree than healthy controls. This result is in line with the vast literature on social impairments in schizophrenia patients (Green et al., 2015; Owen et al., 2016) and more recently on imitation deficits (Matthews et al., 2013; Walther et al., 2015, 2016; Raffard et al., 2018).

Socio-motor synchronization revealed new and interesting results. Healthy controls performance was not significantly different in the three conditions whereas schizophrenia patients showed increasing socio-motor synchronization score as a function of their leading role in the game (Participant leader > Joint improvisation > Confederate leader). Even if the *post hoc* decomposition of this Group × Condition interaction failed to reveal a significant group difference in the Participant leader condition, it is unusual in the literature to observe such high level of socio-motor synchronization in schizophrenia patients (e.g., Kupper et al., 2010; Varlet et al., 2012; Raffard et al., 2015, 2018). Whereas most of studies using the mirror game studied dyads of novices or experts, one originality of our protocol was to face participants with an expert improviser (see Brezis et al., 2017 for a similar procedure). The inner goal of such a procedure was to ensure that changes in socio-motor improvisation and socio-motor synchronization could only be due to changes in participants’ ability to reveal improvised and synchronized motions during the mirror game. We therefore performed a correlational analysis between socio-motor improvisation and synchronization in order to better understand the trade-off between improvisation and synchronization. Results revealed that socio-motor improvisation negatively correlates with socio-motor synchronization for both groups in all conditions, demonstrating the contradiction between improvising and being synchronized. However, despite the evidence that healthy controls show higher socio-motor improvisation than schizophrenia patients, they synchronize equally in the three conditions whereas schizophrenia patients synchronize better when they are leaders rather than sharing the leadership or followers. First of all, these results explain why schizophrenia patients exhibit such high level of synchronization when they are leader in our protocol: because the degree of improvisation is significantly lower than in the control group. However, this low degree of improvisation is also the same in the Joint improvisation and Confederate leader conditions, without giving rise to high levels of synchronization. The second main result of this correlational analysis is thus to show that when the patients lead, the expert follow easily and the dyad socio-motor synchronization is high; however, when the expert leads, patients fail to follow easily, and the dyad socio-motor synchronization is low (Figure 3). Considering the negative correlation between socio-motor improvisation and synchronization, the astonishing good synchronization for schizophrenia patients leading the game is thus explained by the combination of a low degree of improvisation (motions highly predictable) and high skills of the expert as a follower.

Finally, executive measures that have been largely associated with flexibility and *cognitive* improvisation (Sass, 2001) were impaired in schizophrenia patients compared to healthy controls (Table 1). Our study also revealed neither a correlation between socio-motor improvisation and executive measures (Appendix Table 1) nor a correlation between socio-motor improvisation and symptomatology (Table 2). No significant correlations were found between neurological soft signs nor anti-psychotic treatments (mean dose of chlorpromazine equivalents). Socio-motor improvisation impairments are not secondary to cognitive functioning neurological soft signs, nor anti-psychotics treatments and thus can be considered as abnormalities mainly related to motor synchrony deficits. Altogether, the results show that socio-motor improvisation deficits in schizophrenia depends principally on synchronization skill ability. This might be interpreted as a dissociation between socio-motor improvisation and *cognitive* improvisation, i.e., that being able to improvise at the motor level in a social context does not depend on the cognitive ability to generate new ideas. Altogether, our results suggest that socio-motor improvisation and *cognitive* improvisation do not share the same underlying processes and provide a direct evidence that being able to improvise in individual situations is fundamentally different than being able to improvise in a social context. Therefore, our study explains why mental illness is associated with creativity in one-to-many situations (“creative minds,” “mad genius”) more than in collaborative situations such as brainstorming in a company or team activities.

## Limits

The scale chosen to measure negative symptoms (PANSS) was outdated and could explain the lack of association between the severity of negative symptoms and performance in the socio-motor improvisation task. The use of more specific and sensitive scales for negative symptoms such as the Brief Negative Symptom Scale BNSS (Kirkpatrick et al., 2011) or the clinical Assessment Interview for Negative Symptoms (CAINS, Kring et al., 2013) could have been a better choice in a study that evaluated clinical constructs which are akin to and overlap with the concept of negative symptoms of schizophrenia. However, neither the CAINS nor the BNSS were validated in French at the time of our study. This is nevertheless an important limit of the present study.

Another limit of this study relates to the low symptom severity of the patients group. Indeed, contrary to several past studies demonstrating that patients with schizophrenia are truly impaired when imitating hand gestures, which was linked to social functioning, motor abnormalities (Matthews et al., 2013; Walther et al., 2015, 2016) and negative symptoms (Kupper et al., 2010; Lavelle et al., 2013). In the present study, the PANSS scores reflected a hardly symptomatic sample of patients with schizophrenia which could partly explain the lack of associations found in the present study between psychotic symptoms and the socio-motor improvisation task. Future studies including samples with higher levels of negative and motor symptoms are needed to further investigate the ecological validity of this task.

## Conclusion

We defined socio-motor improvisation as an individual’s ability to respond in a divergent, original or flexible manner to motor challenges induced by social interactions while being still socially efficient. Comparing Schizophrenia patients and healthy controls skills during the game, we found that patients were less able to improvise than healthy controls. We also showed that patients exhibited significantly higher difficulties to being synchronized with someone else when they were followers rather than leaders of the game. Interestingly, such impairments were not correlated with the usual *cognitive* improvisation. Altogether, our results suggest that socio-motor improvisation and *cognitive* improvisation do not share the same underlying processes and provide a direct evidence that being able to improvise in individual situations is fundamentally different than being able to improvise in a social context. Finally, the significant negative correlation exhibited by schizophrenia patients between socio-motor improvisation and socio-motor synchronization suggests that synchronization is an interesting key factor to enhance social skills such as social improvisation and flexibility in a clinical context.

## Data Availability Statement

The raw data supporting the conclusions of this article will be made available by the authors, without undue reservation.

## Ethics Statement

The studies involving human participants were reviewed and approved by CPP Sud-Méditerranée-III, Nîmes, France, #2013.04.05 and DA/2013-13. The patients/participants provided their written informed consent to participate in this study.

## Author Contributions

RS, DC, and SR contributed to the acquisition of funding. RS proposed the original idea, designed the study, developed the technology for the data acquisition, performed the statistical analyses, and wrote the first draft. RS and J-FC recruited and assessed the participants. RS and SR prepared the final manuscript. All authors reviewed the manuscript.

## Conflict of Interest

RS was employed by the company SAS Mooven while writing the last version of this manuscript without competing interests, or other interests that might be perceived to influence the results and discussion reported in this manuscript. The remaining authors declare that the research was conducted in the absence of any commercial or financial relationships that could be construed as a potential conflict of interest.

## Publisher’s Note

All claims expressed in this article are solely those of the authors and do not necessarily represent those of their affiliated organizations, or those of the publisher, the editors and the reviewers. Any product that may be evaluated in this article, or claim that may be made by its manufacturer, is not guaranteed or endorsed by the publisher.
