# Supplementary material for: Socio-Motor Improvisation in Schizophrenia: A Case-Control Study in a Sample of Stable Patients
Source: Front Hum Neurosci. 2021 Oct 21;15:676242. doi: 10.3389/fnhum.2021.676242 (PMC8567989; doi:10.3389/fnhum.2021.676242)
Supplement: Supplementary file 1 [file Data_Sheet_1.pdf]

Appendix: Sociomotor improvisation in schizophrenia: a case-control study in a sample of stable patients

Robin N. Salesse[1,2*], Jean-François Casties[1], Delphine Capdevielle[1,3], and Stéphane Raffard[1,4]

[1]University Department of Adult Psychiatry, Montpellier Hospital, France
[2]CTIsuccess by Mooven, Contract Research Organisation, Montpellier, France
[3]Inserm U1061, Montpellier, FR
[4]Univ Paul Valéry Montpellier 3, Univ. Montpellier, EPSYLON EA, Montpellier, France

Appendix

Correlation between socio-motor and cognitive variables

	Socio-motor Improvisation			Socio-motor Synchronization		
	Participant Leader	Joint Improvisation	Confederate Leader	Participant Leader	Joint Improvisation	Confederate Leader
LoE	-.2732 *p=.160*	-.1170 *p=.553*	-.1728 *p=.379*	.2027 *p=.301*	.0920 *p=.642*	.1721 *p=.381*
fNART	-.2372 *p=.224*	-.0783 *p=.692*	.1053 *p=.594*	.0927 *p=.639*	.0643 *p=.745*	.1098 *p=.578*
Trail Making Test						
TMT A	-.1658 *p=.399*	-.1426 *p=.469*	-.1343 *p=.496*	.2375 *p=.224*	.1738 *p=.376*	-.0903 *p=.648*
TMT B	.2468 *p=.205*	.2439 *p=.211*	.2951 *p=.127*	-.2755 *p=.156*	-.1588 *p=.420*	-.2127 *p=.277*
TMT B-A	.3033 *p=.117*	.2944 *p=.128*	.3467 *p=.071*	-.3515 *p=.067*	-.2118 *p=.279*	-.2034 *p=.299*
Liebowitz Social Anxiety Scale						
Anxiety	-.1986 *p=.311*	-.1551 *p=.431*	-.0637 *p=.747*	.1462 *p=.458*	-.0430 *p=.828*	.0549 *p=.782*
Avoidance	-.2613 *p=.179*	-.2291 *p=.241*	-.0853 *p=.666*	.2384 *p=.222*	.0946 *p=.632*	.1081 *p=.584*
Social Cognition (AQ)	.1548 *p=.432*	.1760 *p=.370*	.0691 *p=.727*	-.1132 *p=.566*	-.2727 *p=.160*	-.1585 *p=.421*
Theory of Mind (ToM15)						
False-belief	-.2241 *p=.252*	.1699 *p=.387*	.0741 *p=.708*	.1060 *p=.592*	-.1673 *p=.395*	.2454 *p=.208*
Understanding	-.4292 *p=.023*	-.4045 *p=.033*	-.1416 *p=.472*	.3312 *p=.085*	**.4963** ***p=.007****	.4485 *p=.017*

Table 1. Correlation between Cognitive creativity and Socio-motor Variables. *r* and *p* values are presented for the correlation between [Level of education, fNART, TMT, Liebowitz Social Anxiety Scale, Social Cognition and Theory of Mind] and [Socio-motor creativity and synchronization] for each condition. Bonferroni's adjustment for 6 comparisons lowers the alpha value from 0.05 to 0.0083, * represents significant correlations < .0083

kinematic characteristics

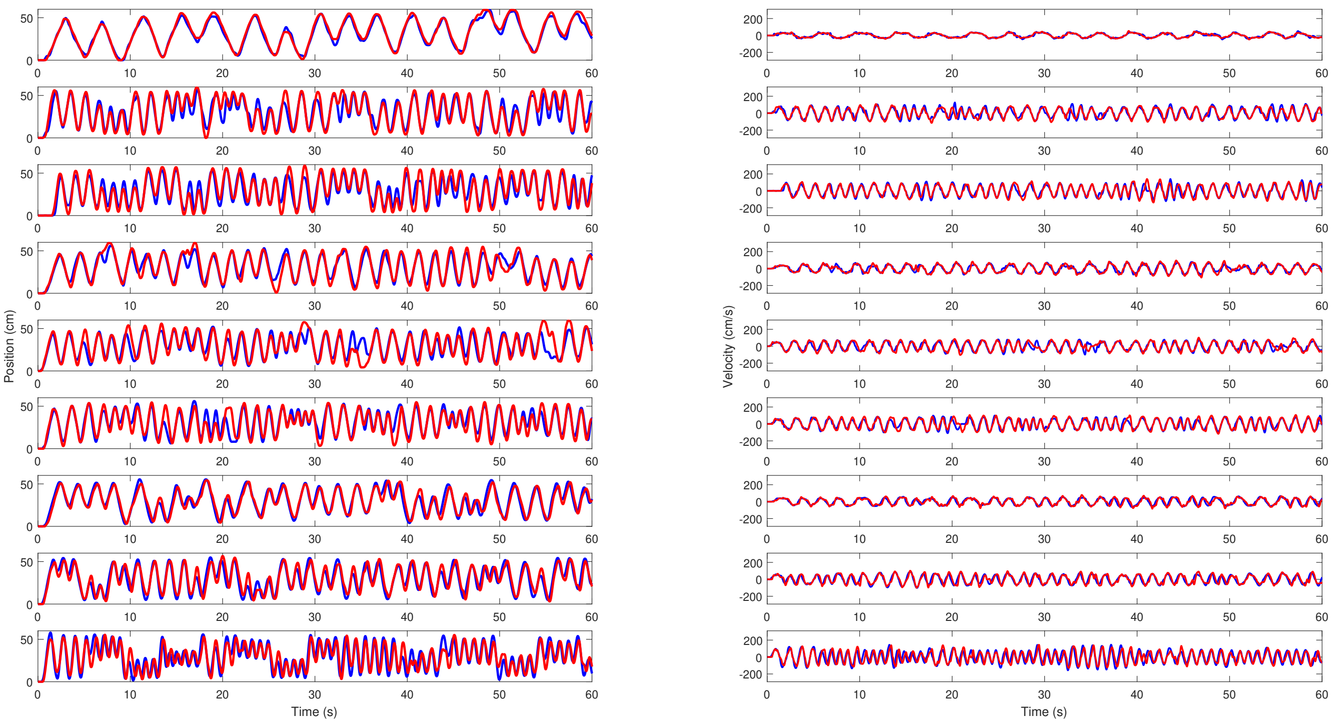

Figure 1. Sample of detailed time series for a participant. Left times series display the position as a function of time. Right time series display the corresponding velocity. Red lines correspond to the participant whereas blue lines correspond to the confederate partner. Time series are presented from top to bottom in order of presentation of the trials, i.e. Participant leader, Confederate leader, Joint improvisation, Confederate leader, Joint improvisation, Participant leader, Joint improvisation, Participant leader, and Confederate leader. The figure shows the different "styles" of motion as a function of the condition. The Joint improvisation condition often displaying a mix of both Participant and Confederate "motion style".

The aim of the two following figures is to allow observing that schizophrenia patients and healthy controls do not differ significantly in terms of kinematic characteristics.

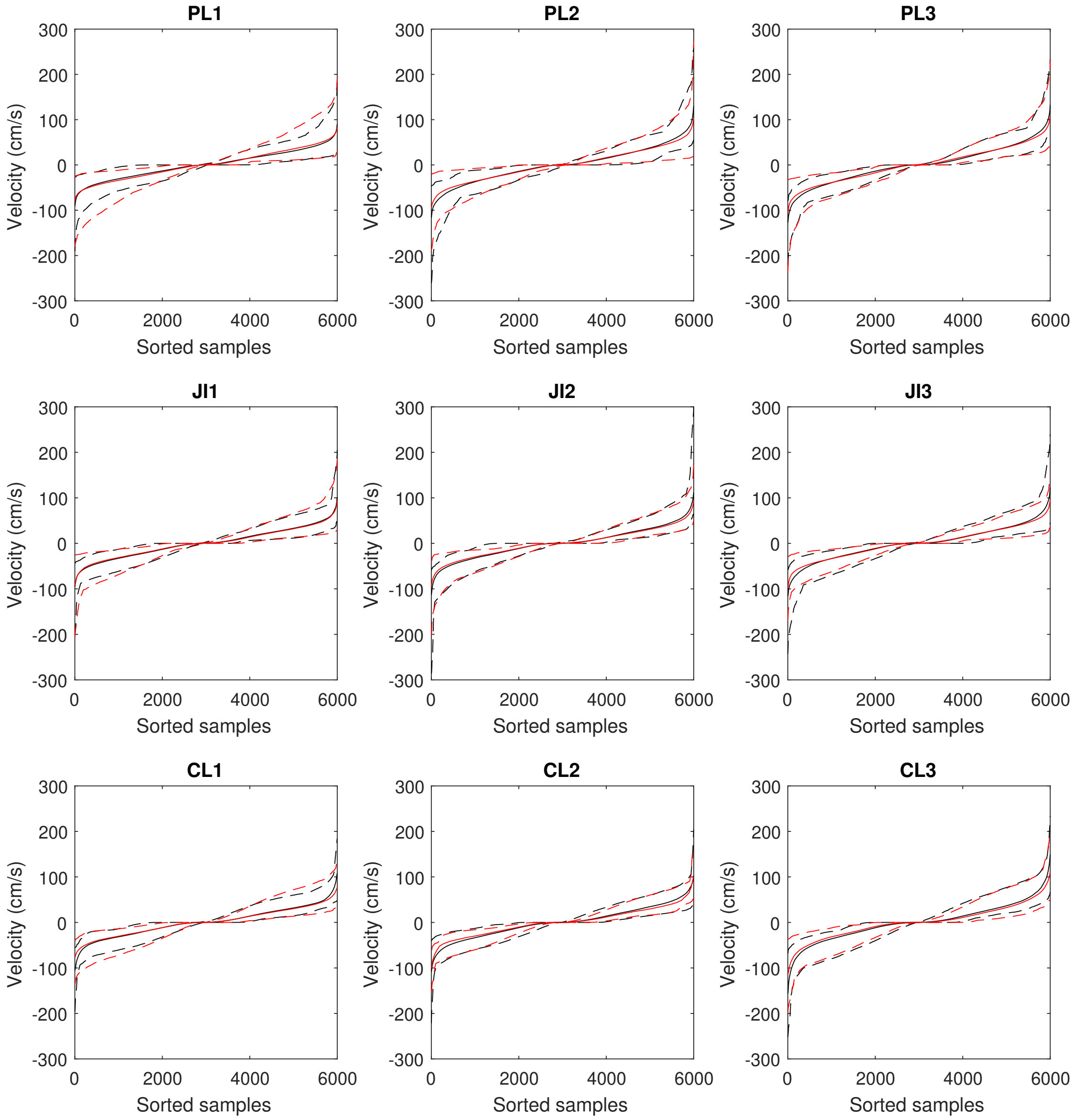

Figure 2. This figure displays the velocity profile for each condition and each group. Titles refers the condition: PL for Participant leader, JI for Joint improvisation and CL for Confederate leader. Numbers following each title refers to the trial for the conditions. Red lines correspond to the Schizophrenia patients and black lines correspond to the Healthy controls. Solid lines represents the mean velocity and dashed lines represents respectively the maximum velocity and the minimum velocity.

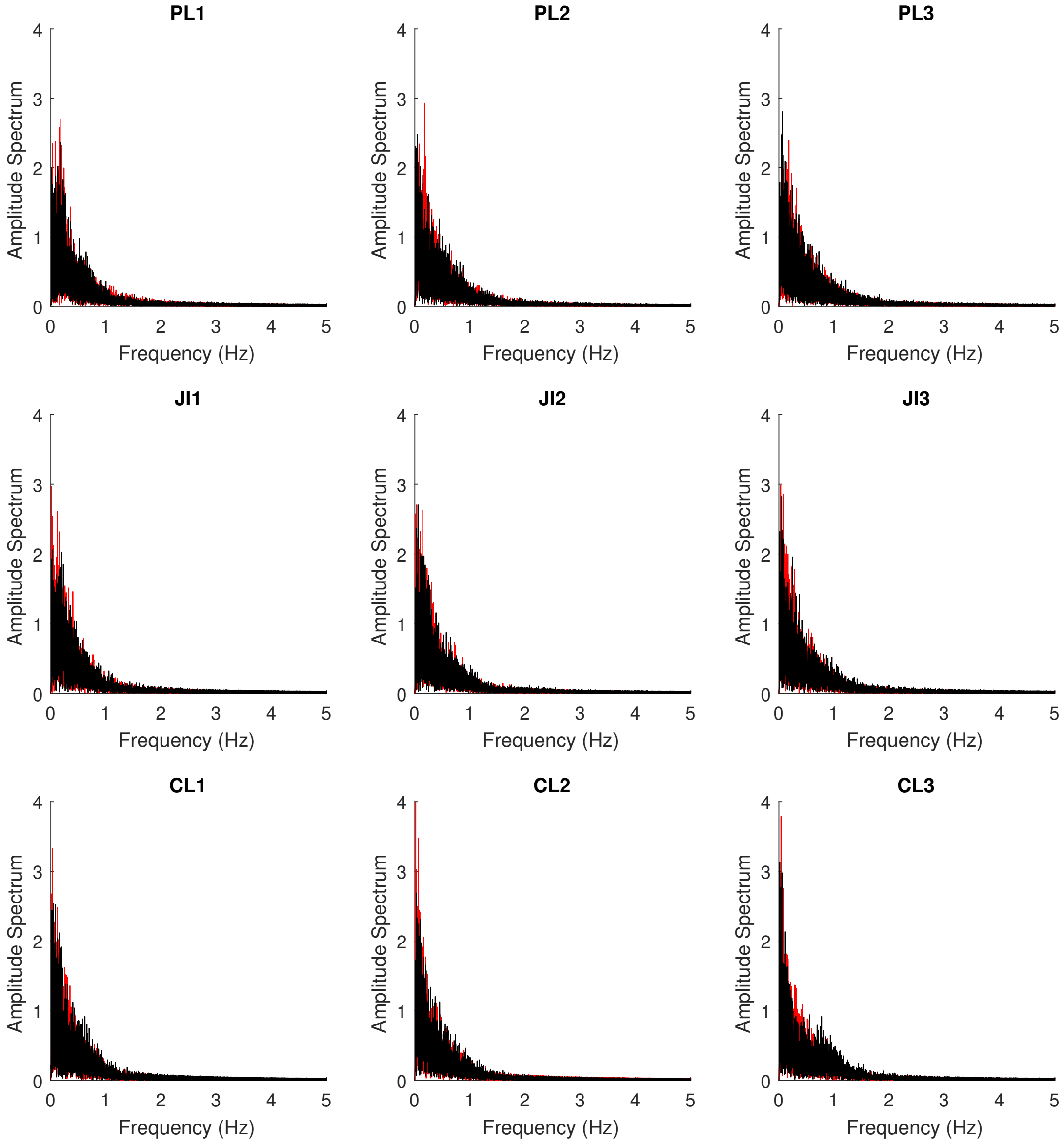

Figure 3. This figure displays the Amplitude spectrum profile for each condition and each group. Titles refers the condition: PL for Participant leader, JI for Joint improvisation and CL for Confederate leader. Numbers following each title refers to the trial for the conditions. Red lines correspond to the Schizophrenia patients and black lines correspond to the Healthy controls.
